# Prevalence of Abnormal Thyroid-Stimulating Hormone (TSH) Levels and Their Association With Iron Status in Pregnancy: A Retrospective Cross-Sectional Study

**DOI:** 10.7759/cureus.96484

**Published:** 2025-11-10

**Authors:** Asma S Al Khofi, Aisha A Bubshait, Latifah A Alhashim, Sarah K Alateeq, Asma M Al Omran, Fatimah N Al Jamaan

**Affiliations:** 1 Department of Family Medicine, Al-Ahsa Health Cluster, Al-Ahsa, SAU; 2 Department of Family Medicine, Eastern Health Cluster, Dammam, SAU

**Keywords:** iron deficiency anemia (ida), kingdom of saudi arabia (ksa), pregnancy-induced thyroid dysfunction, pregnant women, tsh: thyroid-stimulating hormone

## Abstract

Background

Thyroid hormones play a vital role in maternal metabolism and fetal neurodevelopment. Pregnancy causes physiological changes in thyroid function, particularly during the early trimesters, when rising human chorionic gonadotropin levels temporarily lower thyroid-stimulating hormone (TSH) levels before increasing later in pregnancy. Iron deficiency anemia is the most common hematologic disorder during pregnancy, mainly due to hemodilution, poor dietary intake, and short intervals between pregnancies. Because iron is required for thyroid peroxidase activity, its deficiency may lead to thyroid dysfunction.

Methods

A retrospective cross-sectional study was conducted from January 2023 to July 2025 across primary health care centers in Al-Ahsa, Saudi Arabia. A total of 8,542 pregnant women aged 18-40 years with singleton pregnancies and available results for hemoglobin, ferritin, and thyroid function tests were included in the analysis. Women with high-risk conditions or incomplete data were excluded from the study. Data were analyzed using IBM SPSS Statistics for Windows, Version 28 (Released 2023; IBM Corp., Armonk, New York, United States) with chi-square, Pearson’s correlation, and multiple linear regression tests. A p-value less than 0.05 was considered statistically significant. The study was approved by the Institutional Review Board (IRB) of Maternity and Children Hospital, Al-Ahsa.

Results

A total of 8,542 pregnant women were included in the analysis, with a mean age of 29.4 years. Iron deficiency was observed in 76.6% of participants, and anemia was present in 46.2% of pregnant women. Thyroid dysfunction was identified in 2.6% of the cohort, while 97.4% were classified as euthyroid. Low ferritin and hemoglobin concentrations were each significantly associated with elevated TSH levels (p = 0.001 and p = 0.034, respectively), supporting an association between iron deficiency and thyroid dysfunction. No statistically significant associations were observed between thyroid function and either maternal age or glycated hemoglobin levels.

Conclusion

This study demonstrated a significant association between iron deficiency and thyroid dysfunction during pregnancy. Lower ferritin and hemoglobin levels were linked to elevated TSH, suggesting that impaired iron status may contribute to altered thyroid regulation. These findings highlight the importance of concurrent screening for iron deficiency and thyroid function in early pregnancy to improve maternal and fetal health outcomes.

## Introduction

The thyroid gland is an essential part of the endocrine system for metabolism, homeostasis, growth, and cellular differentiation regulation [1]. Its hormones play a significant role in fetal development throughout the pregnancy. During the first trimester, a short-term surge of free thyroxine (FT4) levels happens under the influence of hCG elevation, which results in a decline in thyroid-stimulating hormone (TSH) concentrations. While in the second trimester, the FT4 levels diminished, mirrored by an augmentation in TSH levels that continued to expand gradually until term [2]. These physiological shifts, accompanied by other factors such as iodine deficiency and autoimmunity, can predispose some women to develop hypothyroidism. Globally, hypothyroidism is the most prevalent endocrine disorder, affecting three to five percent of pregnant women. This condition can adversely impact fetal intelligence, contribute to premature birth, fetal malformation, pregnancy loss, and lead to other complications [3,4].

Anemia is defined as a hemoglobin concentration of less than 12 g/dl in non-pregnant women. For pregnant women, the World Health Organization (WHO) sets trimester-specific thresholds: less than 11 g/dl in the first and third trimesters, while in the second trimester, less than 10.5 g/dl. IDA, which represents 75% of cases considered the most prevalent cause of anemia in pregnancy due to physiologic expansion of blood that results in hemodilution [5]. Causes include inadequate dietary intake and iron depletion before pregnancy due to menstruation or a previous pregnancy less than two years ago [6].

Multiple studies showed a relationship between IDA and hypothyroidism. The iron is stored predominantly as ferritin, which is an intracellular protein. Patients with thyroid disorders often exhibit variations in serum ferritin levels. Likewise, fluctuations in thyroid hormone concentrations are observed in response to changes in serum ferritin. Additionally, serum ferritin levels tend to rise following the initiation of treatment for hypothyroidism [6]. For example, a study done in China for first-trimester pregnant women found that patients with anemia have significantly higher TSH levels compared with non-anemic peers [7]. Another study conducted in Iraq spanning the second and third trimesters showed that inadequate maternal iron status has been associated with elevated TSH levels and reduced FT4 concentrations [6]. Locally, a study conducted in Jeddah found that 40% of patients with hypothyroidism had microcytic, hypochromic anemia [8].

A comprehensive evaluation of both conditions is vital and significantly impacts the pregnancy outcome. Thyroid function tests (TFTs), which include measurements of TSH, FT4, and free triiodothyronine (FT3), are routinely used for this purpose. When necessary, we may conduct additional assessments like antithyroid peroxidase (anti-TPO) antibody testing to identify autoimmune thyroid conditions [3]. Serum ferritin is widely employed in both clinical settings and research as a standard indicator of IDA during pregnancy. Nonetheless, its diagnostic accuracy is limited, as ferritin is influenced by systemic inflammation, infectious processes, and the physiological plasma volume expansion during gestation. In contrast to ferritin, soluble transferrin receptor (sTfR) concentrations remain largely unaffected by chronic inflammatory states, which makes it a more specific biomarker for distinguishing between IDA and anemia of chronic disease (ACD). Notably, sTfR levels typically increase in cases of IDA but remain stable in isolated ACD, thus providing diagnostic clarity in patients with overlapping etiologies of anemia [6]. 

To date, there are no published studies done in our region to examine the changes of TSH during the early pregnancy in anemic and non-anemic mothers, implying a large sample size from primary health care visitors. The purpose of this study is to identify a critical gap in the literature concerning thyroid dysfunction between iron-deficient and non-iron-deficient pregnant women, as well as to assess the correlation between different iron status indicators and thyroid hormone levels across pregnancy trimesters, determining the proportion of iron-deficient pregnant women who develop thyroid dysfunction, whether subclinical or overt. A better understanding of these associations may enhance screening strategies and improve maternal-fetal health outcomes.

## Materials and methods

This retrospective cross-sectional study was conducted from January 2023 to July 2025 in PHCs across Al-Ahsa, Saudi Arabia. A total of 18,828 electronic health records of pregnant women were initially reviewed. All eligible cases that met the inclusion and exclusion criteria during the study period were included in the analysis. After applying the eligibility criteria, 8,542 women were included in the final analysis.

Eligible women were aged 18-40 years, were in their first antenatal care visit (ANC) with singleton pregnancies, and had a complete blood count, serum ferritin, TSH, and glycated hemoglobin (HbA1c) performed during the same encounter. Women were excluded if they were younger than 18 or older than 40 years of age, had high-risk conditions such as multiple pregnancies, hemolytic anemia, autoimmune diseases, or chronic systemic disorders that are routinely referred to secondary care hospitals, or if any blood tests (hemoglobin, ferritin, TSH, or HbA1c) were missing.

Normal cut-off values were defined as follows: hemoglobin above 11 g/dL was classified as normal, mild anemia was defined as 10.0-10.9 g/dL, moderate anemia as 7.0-9.9 g/dL, and severe anemia as below 7.0 g/dL [5]. Serum ferritin above 30 ng/mL was considered normal, with iron deficiency defined as below 30 ng/mL [9]. Thyroid dysfunction was defined as TSH levels below 0.1 mIU/L or above 2.5 mIU/L in the first trimester [10].

Medical and demographic data (maternal age) and laboratory data (Hb, ferritin, serum iron, TIBC, TSH, FT3, FT4) were extracted from electronic health records. A standardized data collection form was used, and all datasets were checked for completeness, consistency, and accuracy.

Data were analyzed using IBM SPSS Statistics for Windows, Version 28 (Released 2023; IBM Corp., Armonk, New York, United States). Continuous variables were first checked for normality. Categorical variables were summarized as counts and percentages, while continuous variables were presented as means with standard deviations or as medians with interquartile ranges, depending on their distribution. To study associations between thyroid function and maternal characteristics, the Pearson chi-square test was used. Correlations between TSH and other laboratory parameters were assessed using Pearson’s correlation. Multiple linear regression analysis was then applied to identify the factors most strongly influencing TSH levels. A p-value less than 0.05 was considered statistically significant.

The study was reviewed and approved by the Institutional Review Board (IRB) of Maternity and Children Hospital, Al-Ahsa, Saudi Arabia. As anonymized retrospective electronic health record data were used, informed consent was waived. All procedures complied with national regulations and the principles of the Declaration of Helsinki.

## Results

The study population was relatively young, with a mean age of 29.4 years, and the majority (52.6%) were between 26 and 35 years of age. Regarding glycemic status, HbA1c ranged from 1.1 to 9.52% with a mean level of 4.9 ± 1.3%. Iron status revealed a high burden of deficiency: the majority of the study participants (76.6%) had abnormally low serum ferritin levels (less than 30 ng/mL), suggesting a high prevalence of iron deficiency within the sample. In contrast, only 23.4% of individuals exhibited normal ferritin levels (above 30 ng/mL). This pattern was similar in hemoglobin levels, where anemia (below 11 g/dl) was present in 46.2% of participants, with a mean hemoglobin of 11.1 g/dl (Table 1). 

**Table 1:** Bio-Demographic Characteristics of the Study Women During the First Visit of Pregnancy (n = 8,542) HbA1c: Glycated hemoglobin

Data	No (%)
Age in years	
18-20	539 (6.3%)
21-25	1914 (22.4%)
26-30	2326 (27.2%)
31-35	2169 (25.4%)
36-40	1592 (18.6%)
Mean ± SD	29.4 ± 5.8
HbA1c level	
Range	1.1-9.52
Mean ± SD	4.9 ± 1.3
Serum ferritin level (ng/mL)	
Abnormal level (<30 ng/mL)	6545 (76.6%)
Normal level (> 30 ng/mL)	1995 (23.4%)
Mean ± SD	24.1 ± 33.4
Median (IQR)	15.1 (8.3-28.5)
Hemoglobin level	
Anemia (<11 g/dl)	3942 (46.2%)
Normal (> 11 g/dl)	4598 (53.8%)
Mean ± SD	11.1 ± 1.3

Among a cohort of 8,542 pregnant women assessed during their initial antenatal visit, the prevalence of abnormal thyroid profiles was relatively low. The data reveal that the vast majority (97.4%) were classified as euthyroid, indicating normal thyroid function. In contrast, only 2.6% had an abnormal thyroid profile. The thyroid profile values among the study participants showed a wide range, with levels varying from 0.01 to 164.33. The mean value was 2.19 with a standard deviation of 4.94. 

Table 2 shows the association between maternal age, laboratory findings, and thyroid profile. There was no significant association between maternal age and abnormal thyroid profile (p = 0.752), as the percentage of thyroid abnormalities remained low and consistent across all age groups, ranging from 2.4% to 3.3%. In contrast, serum ferritin levels showed a significant association with abnormal thyroid profile (p = 0.001). Women with normal ferritin levels (above 30 ng/mL) had a notably higher prevalence of abnormal thyroid profile (4.3%) compared to those with abnormal ferritin levels (<30 ng/mL), where the dysfunction rate was only 2.1%. The hemoglobin level was also significantly associated with abnormal thyroid profile (p = 0.034), with anemic women (below 11 g/dL) having a higher rate of thyroid abnormalities (3.0%) compared to non-anemic women (2.3%). Finally, HbA1c did not show a significant difference between the euthyroid and abnormal thyroid profile groups (p = 0.847). 

**Table 2:** Association Between Maternal Age, Laboratory Findings, and Thyroid Profile During the First Visit of Pregnancy (n = 8,542) P: Pearson X2 test; $: Independent samples t-test; *p < 0.05 (significant); HbA1c: Glycated hemoglobin

Factors	Thyroid Profile		p-value
	Euthyroid	Abnormal Thyroid Profile	
	No (%)	No (%)	
Age in years			.752
18-20	521 (96.7%)	18 (3.3%)	
21-25	1865 (97.4%)	49 (2.6%)	
26-30	2267 (97.5%)	59 (2.5%)	
31-35	2117 (97.6%)	52 (2.4%)	
36-40	1546 (97.1%)	46 (2.9%)	
Serum ferritin level (ng/mL)			.001*
Abnormal level (<30 ng/mL)	6406 (97.9%)	139 (2.1%)	
Normal level (> 30 ng/mL)	1910 (95.7%)	85 (4.3%)	
Hemoglobin level			.034*
Anemia (<11 g/dl)	3823 (97.0%)	119 (3.0%)	
Normal (> 11 g/dl)	4493 (97.7%)	105 (2.3%)	
HbA1c			.847^$^
Range	1.1-9.52	1.3-9.4	
Mean ± SD	4.9 ± 1.3	5.0 ± 1.0	

Table 3 reveals the correlation analysis in several statistically significant associations between maternal factors and thyroid profile. Maternal age showed a weak but positive correlation with the thyroid profile (r = 0.029, p = 0.007), suggesting that advancing age slightly increased the likelihood of an abnormal thyroid profile. HbA1c% showed a negative, insignificant correlation (r = -.015, p = 0.227). On the contrary, both serum ferritin (r = -0.070, p = 0.049) and hemoglobin levels (r = -0.024, p = 0.029) showed weak negative correlations, indicating that lower iron storage and reduced hemoglobin were linked with altered thyroid function. 

**Table 3:** Correlation Between Maternal Age, Laboratory Findings, and Thyroid Profile During the First Visit of Pregnancy (n = 8,542) r: Pearson correlation coefficient; *p < 0.05 (significant); HbA1c: Glycated hemoglobin

Items	Thyroid Profile	
	r	p-value
Age in years	.029	.007*
HbA1c%	-.015	.227
Serum ferritin level (ng/mL)	-.070	.049*
Hemoglobin level	-.024	.029*

Table 4 shows the analysis of multivariate linear regression, which reveals several factors associated with TSH levels among pregnant women during their first visit (n = 8,542). Age shows a small positive coefficient (B = 0.02), suggesting a slight increase in the thyroid profile with advancing age, although this association is not statistically significant (p = .087). HbA1c% has a negative coefficient (B = −0.07), indicating a potential inverse relationship with the thyroid profile, but again, the result is not significant (p = .252). In contrast, the serum ferritin level (B = 0.01) and hemoglobin level (B = −0.08) are both significantly associated with the thyroid profile (p = .048 and .014, respectively). Interestingly, while ferritin shows a positive unstandardized coefficient, its standardized beta is negative (−0.026), implying a subtle inverse effect when accounting for variable scales. 

**Table 4:** Multivariate Linear Regression Analysis of Factors Associated With the Thyroid Profile Among Pregnant Women During the First Visit (n = 8,542) B: Regression coefficient; SE: Standard error; * p < 0.05 (significant); HbA1c: Glycated hemoglobin

Items	Unstandardized Coefficients		Standardized Coefficients	t	p-value
	B	SE	Beta		
(Constant)	2.14	.541		4.0	.001
Age in years	0.02	.013	.024	1.7	.087
HbA1c%	-0.07	.059	-.016	-1.1	.252
Serum ferritin level (ng/mL)	0.01	.002	-.026	-2.0	.048*
Hemoglobin level	-0.08	.060	-.018	-2.3	.014*

Table 5 presents the correlation between TSH levels and selected biochemical parameters, HbA1c%, serum ferritin, and hemoglobin, across different age groups among pregnant women. The findings reveal age-specific variations in the strength and direction of associations. In the youngest group (18-20 years), TSH shows a significant negative correlation with serum ferritin (r = −.104, p = .016), suggesting that lower iron storage may be linked to elevated TSH. Among women aged 21-25, serum ferritin again demonstrates a stronger negative correlation with TSH (r = −.144, p = .001), while HbA1c% and hemoglobin show no significant associations. This trend continues in the 26-30 and 36-40 age groups, where serum ferritin maintains a significant inverse relationship with TSH (r = −.066 and −.065, respectively), and hemoglobin shows a modest negative correlation in the oldest group (r = −.053, p = .035). In contrast, the 31-35 age group exhibits no significant correlations between TSH and any of the biochemical markers. 

**Table 5:** Correlation Between TSH and Selected Biochemical Parameters Across Different Age Groups Among Pregnant Women r: Pearson correlation coefficient; TSH: Thyroid-stimulating hormone; HbA1c: Glycated hemoglobin; * p < 0.05 (significant); ** p < 0.01 (high significance)

Age Group	Variable	TSH	
		r	p-value
18–20 years	HbA1c%	.061	0.290
	Serum Ferritin	–.104*	0.016
	Hemoglobin	.105*	0.015
21–25 years	HbA1c%	.007	0.804
	Serum Ferritin	–.144**	0.001
	Hemoglobin	–.025	0.265
26–30 years	HbA1c%	.003	0.924
	Serum Ferritin	–.066**	0.001
	Hemoglobin	–.006	0.787
31–35 years	HbA1c%	-.033	0.241
	Serum Ferritin	–.038	0.078
	Hemoglobin	0.025	0.245
36–40 years	HbA1c%	.017	0.596
	Serum Ferritin	–.065**	0.010
	Hemoglobin	–.053*	0.035

## Discussion

Thyroid hormones play a vital role during pregnancy, influencing maternal well-being, fetal brain development, and overall pregnancy outcomes. Physiological changes that occur during pregnancy affect thyroid hormone production and often result in lower serum TSH concentrations [11]. As a result, pregnant women often exhibit lower serum TSH levels compared with non-pregnant women. Globally, thyroid dysfunction affects approximately five to seven percent of pregnancies [12]. Among these disorders, spontaneous hypothyroidism is the most common, occurring in two to three percent of pregnant women, whereas hyperthyroidism is less frequent, with a prevalence of 0.1-0.4% [13]. A recent systematic review and meta-analysis have reported a notably higher prevalence across Arab countries, with rates reaching 27% for overt hypothyroidism, 20% for subclinical hypothyroidism, and less than 2% for hyperthyroidism [14]. In Saudi Arabia, the overall prevalence of thyroid disease remains uncertain. However, reports from the general population suggest rates ranging between 18.7% and 25.5% [15]. Among pregnant women, a study from Riyadh identified a 13% prevalence of thyroid dysfunction [16]. In contrast, the current study found a considerably lower prevalence of abnormal thyroid profiles, at only 2.6%.

Several maternal factors can influence thyroid function during pregnancy. In the present study, age-related variations in thyroid profiles were observed, although this association did not reach statistical significance. This pattern is consistent with prior studies showing that thyroid dysfunction tends to increase with advancing maternal age [17]. In contrast, other reports found no significant association between maternal age and autoimmune thyroid disease during pregnancy, consistent with the current study's findings [18].

Beyond maternal age, well-established risk factors for thyroid dysfunction include female sex, pregnancy itself, comorbidities such as diabetes mellitus and obesity, as well as a family history of thyroid disease [19]. Although the current study did not demonstrate a statistically significant correlation between HbA1c and TSH levels, the biological connection between diabetes and thyroid dysfunction is well recognized [20]. Furthermore, previous studies have identified associations between gestational diabetes, altered thyroid function, and an increased prevalence of thyroid autoantibodies during pregnancy [21,22]. Given the high prevalence of both thyroid disorders and diabetes in Saudi Arabia, our findings emphasize the importance of re-evaluating thyroid screening practices in early pregnancy to ensure timely detection and management.

In addition to thyroid-related findings, the present study also investigated iron status during pregnancy, recognizing its close physiological link with thyroid function. During pregnancy, the demand for iron increases substantially to support fetal growth and maternal blood volume, which may contribute to various health problems, including anemia [23]. The current study highlights the high percentage of iron deficiency and anemia among pregnant women in the Al-Ahsa region. It shows that 76.6% of them have low serum ferritin levels, and 46.2% are diagnosed with anemia. These rates are notably higher than those reported in both regional and global literature.

A national systematic review published in 2023 reported that anemia prevalence among pregnant women in various cities across Saudi Arabia ranges from 18% to 58% [24]. In contrast, neighboring Arab countries showed varied rates, with Egypt closely matching the current study's finding at 49%, while Jordan and Sudan recorded lower prevalence rates of 27.4% and 37%, respectively [23]. Similarly, the WHO estimated that, by 2023, around 35.5% of pregnant women globally will have anemia, mostly due to iron deficiency [25]. This emphasizes that anemia during pregnancy remains a common public health concern in many countries.

Despite the high burden of iron deficiency, a notable finding in the current study was that pregnant women with normal serum ferritin levels (>30 ng/mL) had a higher rate of abnormal thyroid function. Specifically, the rate of abnormal thyroid function in this group was 4.3%, compared to those with low ferritin levels. Interestingly, this finding contrasts with the known medical association between ferritin and TSH, as numerous studies have shown that reduced ferritin, reflecting iron deficiency, is commonly linked to elevated TSH and an increased risk of hypothyroidism [26]. For example, a study from Northern Saudi Arabia reported that 60.3% of anemic women and 49.3% of those with iron deficiency were hypothyroid. Thus, this supports the negative association between low iron storage and thyroid changes [27].

The elevated ferritin levels observed in the study population may be partly attributable to the high prevalence of hemoglobinopathies, such as sickle cell disease and thalassemia, in our region. Moreover, ferritin can be elevated due to non-iron-related factors, including chronic inflammation, oxidative stress, and prior blood transfusions. As a result, these mechanisms may obscure true iron deficiency and complicate the interpretation of its association with thyroid function [28]. Consistently, several recent studies have reported no significant correlation between ferritin and TSH, showing that other factors can make this link between iron status and thyroid dysfunction difficult to interpret [29].

In addition, the current study identified a weak but significant inverse relationship between hemoglobin levels and TSH in pregnant women. This corresponds to the previous literature showing that anemia is linked with elevated TSH and potential thyroid dysfunction [26,29]. Furthermore, recent systematic reviews and meta-analyses suggest hemoglobin is a key marker for thyroid changes during pregnancy. This is likely due to its critical role in oxygen transport, which is necessary for thyroid hormone synthesis [29]. Overall, these findings suggest that hemoglobin, rather than ferritin, may serve as a more reliable marker for thyroid dysfunction risk during pregnancy. This supports the inclusion of Hb monitoring as a routine part of antenatal screening.

Strengths, limitations, and clinical importance for future research

The present study provides a large sample size, beginning with 18,828 records, and eventually analyzing 8,542 pregnancies after applying the exclusion criteria. Focusing on the first trimester, low-risk cases, combined with direct electronic laboratory data from the initial ANC visits, minimizes the likelihood of human transcription errors. Variables such as CBC, ferritin, TSH, HbA1c, and maternal age allowed for a comprehensive assessment of iron status and thyroid function during a well-defined gestational period, which supports the reliability and importance of the findings.

Nevertheless, some limitations must be acknowledged: a single measurement was obtained per participant, and the lack of medical history (such as pre-existing chronic diseases and the use of medications) may introduce unknown confounding factors. However, the exclusion of high-risk pregnancies helped in reducing this source of bias. In addition, a complete thyroid profile with autoantibodies was not available, preventing the classification and differentiation of various types of thyroid dysfunction.

Future research could build on these results by following the participants through the whole period of pregnancy, including high-risk pregnancies. Also, they may consider additional clinical and nutritional factors for a better understanding of the link between abnormal TSH and iron status. Such studies could help to define optimal hemoglobin thresholds, improve early detection of anemia and thyroid disorders, and guide effective interventions to enhance maternal and fetal health.

## Conclusions

This study identifies a significant association between iron deficiency and thyroid disorders in pregnant women in Al-Ahsa, Saudi Arabia, indicating that nutritional challenges persist during pregnancy. Lower ferritin and hemoglobin levels were linked to higher TSH levels. However, this association does not establish causality, and further research is needed to determine whether poor iron status directly affects thyroid function. Overt thyroid dysfunction was uncommon (2.6%), but subclinical changes suggest iron deficiency may be linked to early thyroid alterations during pregnancy, though causality remains unproven. No significant association was found between thyroid dysfunction and maternal age or glycemic status (HbA1c). These findings indicate a possible metabolic interaction between iron and thyroid physiology rather than a direct causal relationship. These results highlight the importance of integrated antenatal screening for both iron storage and thyroid function, especially in the first trimester. Early detection and treatment of iron deficiency may help to prevent thyroid disturbances and support maternal and fetal health. Further prospective and interventional studies are needed to clarify causal mechanisms and assess the impact of combined nutritional and hormonal management on pregnancy outcomes.
